# Measurement of cerebrospinal fluid formation and absorption by ventriculo-cisternal perfusion: what is really measured?

**DOI:** 10.3325/cmj.2014.55.317

**Published:** 2014-08

**Authors:** Darko Orešković, Marijan Klarica

**Affiliations:** 1 Ruđer Bošković Institute, Department of Molecular Biology, Zagreb, Croatia; 2 Department of Pharmacology and Croatian Institute for Brain Research, University of Zagreb School of Medicine, Zagreb, Croatia; * Both authors equally participated in the study.

## Abstract

The generally accepted hypothesis on cerebrospinal fluid (CSF) hydrodynamics suggests that CSF is actively formed mainly by choroid plexuses, circulates unidirectionally along the brain ventricles and subarachnoid space, and is passively absorbed mainly into the dural venous sinuses. CSF formation rate (V_f_) has been extensively studied using the ventriculo-cisternal perfusion technique and the results have been used as the key evidence confirming the mentioned hypothesis. This method and the equation for V_f_ calculation are based on the assumption that the dilution of the indicator substance is a consequence of the newly formed CSF, ie, that a higher CSF formation rate will result in a higher degree of dilution. However, it has been experimentally shown that the indicator substance dilution inside the CSF system does not occur because of a “newly formed” CSF, but as consequence of a number of other factors (departure of substances into the surrounding tissue, flowing around the collecting cannula into the cortical and spinal subarachnoid space, departure into the contralateral ventricle, etc). This technique allows “calculation” of the CSF formation even in dead animals, in an *in vitro* model, and in any other part of the CSF system outside the ventricles that is being perfused. Therefore, this method is indirect and any dilution of the indicator substance in the perfusate caused by other reasons would result in questionable and often contradictory conclusions regarding CSF formation rates.

According to the classic hypothesis of cerebrospinal fluid (CSF) physiology, CSF is actively formed inside the brain ventricles, after which it flows unidirectionally along the subarachnoid spaces (SAS) to be absorbed into the venous sinuses across the arachnoid villi and/or via the paraneural sheaths into the lymphatics (1-8). Thus, CSF physiology is based on three key premises: 1) active CSF formation (secretion; V_f_) inside the brain ventricles mainly by choroid plexuses; 2) passive CSF absorption (V_a_) mostly into the venous sinuses on the brain surface via the villi arachnoidales; and 3) unidirectional CSF flow from the site of formation to the site of absorption. This hypothesis has so far been presented as a proven fact in all textbooks and review articles.

According to the classic hypothesis, it is unquestionable that in physiological conditions CSF secretion and absorption inside the CSF space are balanced:

V_f_ = V_a_ [1]

In other words, the secreted CSF volume (inside brain ventricles) has to be the same as the passively absorbed CSF volume (into the venous sinuses and/or lymphatics). If this is not so, a pathological state may occur (eg, hydrocephalus). The same is true for the CSF flow (circulation; Q_CSF_). Namely, CSF secretion and absorption take place at different CSF system sites, and therefore, the flow rate (Q_CSF_) between these two sites has to be of the same magnitude to keep V_f_ equal to V_a._

V_f_ = Q_CSF_ = V_a_ [2]

Thus, CSF secretion changes should be passively followed by CSF flow and absorption changes, in order to maintain the physiological state inside the CSF system. We can conclude from this that active CSF secretion is the main generator of the CSF circulation, to maintain the physiological CSF volume (9). Therefore, it is extremely important to use a precise and reliable method for CSF formation measurement.

However, based on our experimental results, we have recently seriously brought into question the plausibility of this, a nearly hundred-year-old, classic CSF physiology hypothesis, and have suggested a new one (9-16). According to this new hypothesis, interstitial fluid (ISF) and CSF are created by water filtration through the arterial capillary walls across the entire central nervous system (CNS). At the same time, plasma osmolytes are being accumulated inside the capillaries, which generates osmotic counterpressure crucial for the process of ISF/CSF water absorption into the venous capillaries and postcapillary venules. Thus, we can conclude that osmotic and hydrostatic forces are the main factors in the regulation of ISF-CSF volume. If we have in mind the fluid exchange capacity, it is reasonable to say that the choroid plexuses are less likely to be a relevant site for this process, and that this is probably the role of cerebral and spinal capillaries. A constant substances exchange takes place between the CSF system and the adjacent tissue. This process is under influence of pathophysiological conditions inside the CSF compartments. Therefore, CSF secretion cannot take place in only one of these compartments (brain ventricles), and this is also unlikely to be the case with absorption (predominantly inside the cortical subarachnoidal space). This hypothesis (9-11) has recently been tested by water influx into the CSF in aquaporin-1, aquaporin-4 knockout, and wild-type mice using a newly developed water molecular MRI technique based on JJ vicinal coupling between ^17^O and adjacent protons, and water molecules proton exchange (17). The findings have strongly supported the new hypothesis that water movement within the pericapillary spaces, rather than within choroid plexuses and arachnoid villi, is essential for CSF homeostasis.

The recent understanding of CSF physiology has been developed from the quantitative utilization of the ventriculo-cisternal perfusion method. Since other methods have mainly been abandoned, or are used very rarely (9), the aim of this article is primarily to reevaluate the ventriculo-cisternal perfusion method, which is today the only generally accepted method used for determination of the CSF formation rate (18,19). This method produced experimental results that were used to confirm the classic CSF physiology hypothesis and therefore represent its foundation. The method has been considered to be a precise physiological approach for studying cerebrospinal fluid secretion (18), and Cutler et al (19) have concluded that: *“*Important advances in understanding of cerebrospinal fluid physiology have been made since the introduction of a method for perfusion of the Pappenheimer et al (20). This technique has permitted accurate measurement of both the rate of formation and rate of absorption*….”* Although the method itself still represents the headstone of the classic CSF physiology hypothesis, the question is whether the situation is the same after fifty years, and whether the method can withstand criticism of new scientific results. This question will be thoroughly analyzed further in the article.

## VENTRICULO-CISTERNAL PERFUSION METHOD-

**Figure 1:**
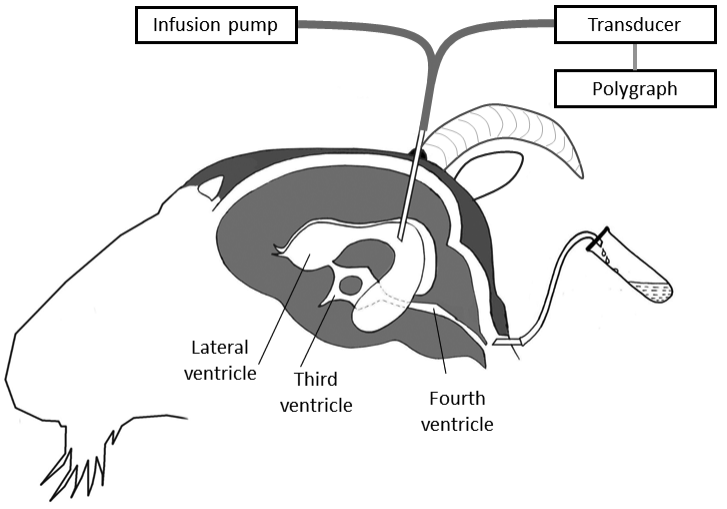
The ventriculo-cisternal perfusion in goats showing the position of the cannula for the infusion of artificial cerebrospinal fluid into the left lateral ventricle and the cannula in the cistern magna for the collection of outflow perfusate.

This method was developed by Pappenheimer et al (20) and Heisey et al (21) on goats, but it has been used on many other experimental animals and in humans. Perfusion is performed from the lateral brain ventricle to the cisterna magna (CM) (ventriculo-cisternal perfusion; Figure 1) using a mock CSF that contains a marker (inulin, albumin, dextran, etc). Determination of CSF formation is based on the assumption that marker dilution in perfusate occurs due to CSF secretion within the brain ventricles (LV), implying that higher CSF secretion increases marker dilution.

The brain ventricles perfusion from the lateral ventricle to the CM can begin after introducing an inflow metal cannula into the lateral brain ventricle, and an outflow cannula into the CM. The inflow cannula is attached to a T-shaped connector, and for the purpose of CSF pressure measurement on the one side connected to a manometer (polygraph) via polyethylene tubing and on the other to a syringe containing artificial fluid with the diluted marker. The syringe is fixed on the perfusion pump that allows the artificial fluid containing the marker (inflow perfusate; V_i_) to flow into the lateral ventricle at a constant perfusion rate (µL/min). The outflow cannula is connected to a plastic tube, through which the outflow perfusate is collected from the brain ventricles and the CM (V_o;_ µL/min_;_ Figure 1). Perfusion is performed under a certain hydrostatic CSF pressure that can be adjusted by setting the end of the outflow polyethylene tubing above (positive pressure) or below (negative pressure) the external auditory channel, whose level is assumed to be a zero value of the hydrostatic CSF pressure.

The method itself is indirect, since the newly formed CSF is not directly measured, but the rate of CSF formation is calculated by marker dilution (for example inulin) in the outflow perfusate. Considering the importance of V_f_ and V_a_ calculation by Heisey et al (21) for CSF physiology, we will cite this part of their article:

“Rate of Formation of CSF in the ventricle, V_f_

Evidence will be presented that diffusion of inulin from the ventricular system is negligible; almost all inulin lost from CSF can be accounted for by bulk absorption distal to the fourth ventricle. It follows that any dilution of inulin during passage through the ventricles results from newly formed inulin-free fluid. Stated mathematically

V_f_ = V_i_ (c_i_ – c_o_)/c_o_ (I)

An alternative form of equation (I) is

V_f_ = C_In_ + V_o_ – V_i_ (II)

Rate of Bulk Absorption of Fluid, V_a_

If net loss of inulin can only occur by bulk absorption distal to the fourth ventricle, then

V_a_ = (V_i_ c_i _– V_o_ c_o_)/c_o_ = clearance of inulin, C_In_ (III)

Clearance of inulin from the CSF system may be compared with glomerular elimination of inulin in the kidney. Renal clearance of inulin is a measure of bulk filtration at the glomerulus; CSF clearance of inulin is a measure of bulk absorption distal to the fourth ventricle,”

where *V* = rate of flow, mL/min; *_i_* and *_o_* = subscript referring to inflow and outflow; *_c_* = concentration, quantity per milliliter; *_f_* and *_a_* = subscript referring to formation and bulk absorption of fluid.

Since the equations were elaborated on the basis of a well-known principle for glomerular filtration quantity calculation using inulin clearance in renal physiology, it is very important to analyze and compare the physiological conditions inside the kidney and CSF system during the use of both methods. The conditions of ventriculo-cisternal perfusion are thoroughly described above. Let us now briefly describe the method for inulin clearance calculation in renal physiology. Inulin is applied intravenously to the patient, who afterwards urinates into a special container. After some time, a certain volume of venous blood is taken as a sample, and urine collection is stopped. Inulin concentration in both blood and urine is calculated, and the volume of collected urine is measured. Glomerular filtration volume is equal to inulin clearance, since all of the glomerular filtrate is cleared from inulin. Therefore, based on the data regarding inulin concentration in the blood, inulin concentration in the urine, volume of collected urine, and the period required for its collection, one can calculate inulin clearance, ie, glomerular filtration (GF). The mathematical equation is the following:

K = V × U/P [3]

where K = plasma clearance; V = volume of urine excreted in minutes (mL/min); U = inulin concentration in plasma (mg/mL); *P* = inulin concentration in urine (mg/mL).

If we compare these two methods, it is crucial to emphasize the following: while calculating inulin clearance (in renal physiology), inulin is being administered intravenously, and after some time the blood sample is collected. In the meantime, the patient spontaneously urinates and urine is collected. Hence, inulin from the blood is transported (filtered) to the kidneys, where it is spontaneously eliminated via urine into the bladder, and afterwards collected into a special kind of canister.

During ventriculo-cisternal perfusion, after animals have been anaesthetized the inflow and outflow cannulas are introduced into the CSF system (Figure 1). Inulin (marker substance diluted in the artificial CSF) is administered into the CSF system using an infusion pump (certain perfusion fluid rate; µL/min), which causes the perfusate to flow (unidirectional circulation) from the inflow cannula positioned inside the lateral ventricle to the outflow cannula positioned inside the CM, finally leading (through a plastic cannula) into the plastic tube (Figure 1). And while for renal inulin clearance calculation, blood circulation and urine excretion (physiological processes) are used, ventriculo-cisternal perfusion calculation is based on the circulation of the artificial CSF through the CSF system, as well as on the outflow perfusate with test substances collected into the plastic tube. This process is a consequence of an infusion pump activity, ie, artificial instead of the natural/physiological CSF circulation is being used. Thus, inulin clearance in renal physiology is a physiological method, while ventriculo-cisternal perfusion is an artificial method. Moreover, such artificial perfusion is in a disaccord with CSF physiology (CSF circulation, but also CSF formation and absorption). In other words, a clearly nonphysiological method has become the generally accepted method for the study of CSF physiology (CSF formation and absorption), which creates a controversy. Furthermore, certain postulates must be satisfied in order to use this method: 1) all CSF must be formed within the ventricles; if any CSF is formed outside of the ventricles, that “outside” CSF will not dilute the marker substance in the outflow perfusate, and the calculated V_f_ will be “falsely” smaller; 2) all CSF must be absorbed outside the ventricles; if any CSF is absorbed within the brain ventricles, the amount of the formed CSF would be reduced by the amount of the CSF absorbed within the ventricles; 3) marker substance cannot be absorbed from the CSF system into the brain tissue and the blood circulation between the inflow and outflow cannula; if it is absorbed, this will lead to marker “dilution,” which will result in a “falsely” higher V_f_; 4) CSF formation must not depend on the perfusion rate and hydrostatic pressure under which perfusion is performed; namely, CSF formation is an active process that should not be affected by these parameters; 5) only a substance that is not present inside the CSF can be used as a marker substance.

It should be stressed that these conditions are based mainly on the classic hypothesis according to which CSF is secreted exclusively inside the brain ventricles, absorbed exclusively on the brain surface outside the ventricles, and circulates between those two spaces. It should also be pointed out that these conditions are taken as indisputable scientific facts, and not as unverified presumptions. Therefore, this represents a scientific discrepancy: parameters which have yet to be confirmed as scientific facts, already exist as ultimate requirements necessary for this method to be used.

Although these considerations put into question the ventriculo-cisternal method, further effort should be invested into the evaluation of the conditions for the CSF formation and absorption calculation. Our attention will primarily be focused on the evaluation of CSF formation (V_f_) calculation, although each evaluation of CSF formation is simultaneously an evaluation of CSF absorption, which is derived from equation [1] we have already discussed:

V_f_ = V_a_ [1]

Using the ventriculo-cisternal perfusion method under physiological conditions, the equation [1] must be:

V_f_ + V_i_ = V_a_ + V_o_ [4]

where V_i_ is the infused volume and V_o_ is collected volume of perfusate, which means that the amount of the CSF that is formed and the amount of infused perfusate must be equal to the amount of the absorbed CSF and the volume of perfusate that is collected in the plastic tube. From equation [4], it could be calculated that:

V_a_ = V_f_ + V_i_ – V_o_ [5]

Thus, equation [5] makes it obvious that if the V_f_ value is incorrect then the V_a_ value also cannot be correct. In other words, incorrect V_f_ values also discredit V_a_ values. The same argument (incorrect V_f_ means incorrect V_a_) can also be applied to the original equations (equation 2 and 3) for CSF absorption calculation by Heisey et al (21). Therefore, let us return to the evaluation of V_f_ calculation by ventriculo-cisternal perfusion method using the equation of Heisey et al (21).

## EVALUATION OF VENTRICULO-CISTERNAL PERFUSION POSTULATES

Although ventriculo-cisternal perfusion method for V_f_ measurement is widely used, assumptions have not been critically and thoroughly evaluated. V_f_ and V_a_ have been calculated in experiments on dogs (22) and on cats (16) by perfusion in isolated brain ventricles. In these experiments, the site of CSF formation (brain ventricles) has been separated from the site of CSF absorption (subarachnoid space), which according to the postulates of this method should show the total amount of secreted CSF (V_f_), but the amount of absorbed CSF (V_a_) should be zero. Namely, as it has been previously mentioned, ventriculo-cisternal perfusion method could be used only if the entire CSF was produced inside the ventricles and absorbed outside of them. However, it was found that, at physiological CSF pressure, only about one third of the total CSF was formed inside the brain ventricles (V_f_ = 16.00 µL/min in dogs and 4.50 µL/min in cats) and that CSF absorption took place within the ventricles as well as in the subarachnoid space. In other words, it is obvious that CSF is also formed outside the ventricles, and that it is also absorbed inside of them. Furthermore, at physiological CSF pressure, the amount of CSF formed inside the ventricles is equal to the CSF amount absorbed inside of them (16), which is in disagreement with the key postulates for the V_f_ and V_a_ calculation using ventriculo-cisternal perfusion method.

Since it was assumed (20,21) that marker substance dilution is a consequence of newly formed CSF, its other possible causes have been disregarded. Furthermore, it is well known that a marker can move from the CSF into the brain parenchyma (5,23-26) and that inulin and other marker substances (27-29) enter rapidly into perivascular CNS spaces, reach a very large surface area of capillaries, and, by slow diffusion across microvascular walls, reach the bloodstream to be rapidly eliminated through urine (30,31). Therefore, an error in the interpretation of the marker dilution degree by absorption into the brain parenchyma will result in false calculation of CSF formation rate.

The calculation of net CSF formation should, according to the classic hypothesis, also be independent from the perfusion rate and intracranial pressure, since V_f_ is considered to be an active and energy-consuming process. CSF secretion should be carried out against the intracranial pressure (ICP), especially because a force weaker than 30 cm H_2_O (the limit within which the experiments were performed) is not sufficient to affect an active process (32). However, during ventriculo-cisternal perfusion with perfusate containing ^3^H-water and blue dextran (m.w. 2 × 10^6^), a hydrostatic pressure increase in the perfusate from negative (-10 cm H_2_O) to positive (+20 cm H_2_O) values had opposite effects on these two substances: increase in blue dextran and decrease in ^3^H-water concentration (26). If we take into account that it was demonstrated that a significant bulk absorption of ^3^H-water occurred within the brain ventricles, all of these losses of the marker substance from the perfusate and changes in marker concentration caused by the loss of water/CSF (since 99% of CSF is water) (14) should result in an incorrect calculation of the net CSF formation. Other studies have also demonstrated that hydrostatic CSF pressure changes cause alterations in CSF formation rate in a way that the pressure increase significantly diminishes the calculated CSF formation (V_f_) (16,19,33-36). Furthermore, although experimental studies on CSF formation have used a wide range of different perfusion rates (21,37), it should be expected that a constant CSF formation (V_f_) rate is calculated. In other words, a change in the perfusion rate should not cause a change in calculated V_f_. However, ventriculo-cisternal perfusion in cats showed that an increase in the perfusion rate (from 32.0 to 65.5, to 125.0, and to 252.0 µL/min) significantly decreased the calculated CSF formation rate (37). These results indicate that different intensity of marker mixing with native CSF at different perfusion rates probably causes a defect in the method (37).

**Figure 2:**
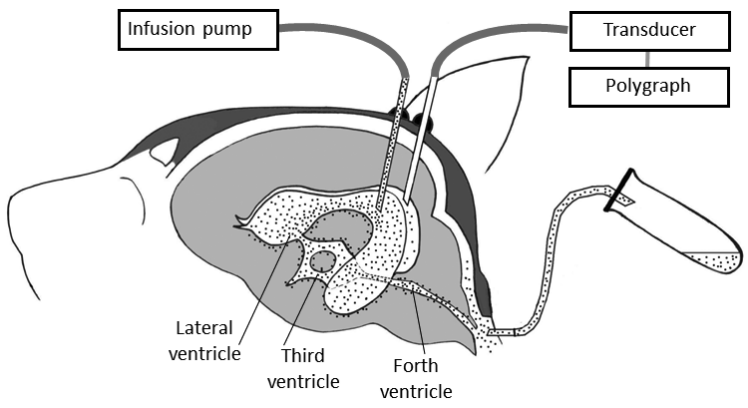
The distribution of marker substances during ventriculo-cisternal perfusion in cats. The black points show an equal marker distribution inside the left lateral, third and fourth ventricle, and a partial distribution in the contralateral ventricle, brain tissue, and cranial and caudal cerebrospinal fluid subarachnoid space about the cisterna magna.

Figure 2 shows the usual distribution of marker substance (for example blue dextran) during perfusion. It can be noticed that there is a mixing problem (the substance is not equally mixed with CSF in all of the perfused parts). A portion of the marker substance goes into the tissue, a portion slowly reaches the contralateral ventricle, and a portion flows around the outflow cannula into the cranial and spinal subarachnoid space, which causes dilution of input perfusate. All of these processes, after a certain perfusion period, result in an almost constant output concentration of marker substance, but with permanent slow marker dilution during the experiment. However, when perfusion conditions such as outflow pressure or rate of perfusion are changed, the marker substance concentration in the outflow perfusate also changes significantly. The interference of hydrostatic CSF pressure and perfusion rate with calculated V_f_ discredits the ventriculo-cisternal perfusion model as a method V_f_ calculation using the equation derived from Heisey et al (21). This makes this method the least precise one. It is necessary to stress that so far no one has determined under which conditions (infusion rate and outflow pressure) ventriculo-cisternal perfusion should be performed. It could be concluded that the postulates necessary for the use of ventriculo-cisternal perfusion method and equation for calculation of CSF formation (V_f_) are not satisfied.

## EXPERIMENTAL CONDITIONS WHICH DISCREDIT THE VENTRICULO-CISTERNAL PERFUSION AS A METHOD FOR DETERMINATION OF CSF FORMATION

Using ventriculo-cisternal perfusion method to measure the CSF formation rate in the rhesus monkey, rate changes have been observed when none were expected (34). The most puzzling finding has been the decline of V_f_ for 4% each hour during the final five hours of perfusion, although the variables known to affect V_f_ remained stable (34). The reason for these unexpected results remains obscure and the reduction may be an artifact of the method.

**Figure 3:**
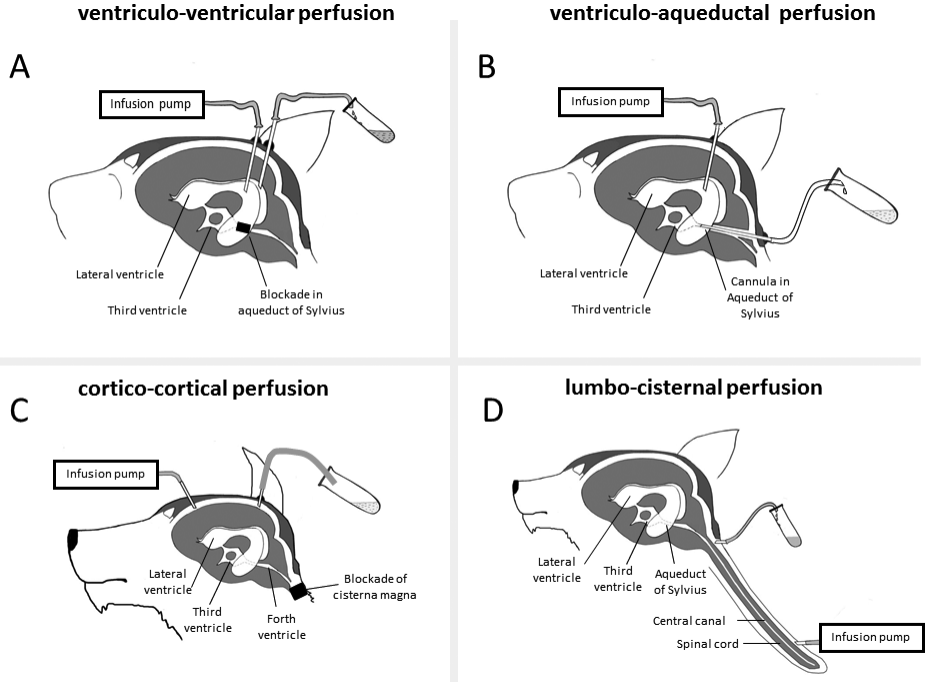
Different types of cerebrospinal fluid system perfusion methods in cats (**A** and **B**) and dogs (**C** and **D**).

Furthermore, since the method is indirect and depends on marker dilution by “newly formed CSF” and since a marker can pass from the CSF into the brain parenchyma (5,23-26), V_f_ should be calculated in any part of the CSF system that will be perfused. Except the most frequent type of ventriculo-cisternal perfusion method, other types of perfusion have been performed in the CSF system of cats and dogs – lateroventricular-lateroventricular, ventriculo-aqueductal, cortico-cisternal, corticofrontal-corticofrontal, cervical-lumbosacral, and lumbosacral-cervical (Figure 3) (15,16,22,38-40), and in each of these models a significant amount of net CSF formation has been calculated. So, we can actually show that CSF is formed inside the ventricles (if we perfuse the ventricles; Figure 3 A and B), but also inside the cortical and spinal subarachnoid space (if we perfuse these parts of the CSF system; Figure 3 C and D). Thus, we can show CSF formation using the equation of Heisey et al (21) anywhere (in whichever segment someone decides to perfuse) because, due to various factors, marker dilution will occur in any case.

In order to evaluate the ventriculo-cisternal perfusion method and V_f_ calculation using the equation from Heisey et al (21), two types of experiments have been done: on animals (sacrificed cats in which anatomical relations were preserved) and on a plastic cylinder, a model that complies completely with all of the postulates necessary for ventriculo-cisternal perfusion. Namely, dextran molecules cannot diffuse into the surrounding space, and they will mix with the fluid inside the cylinder (41). The entire outflow perfusate will be collected (no dextran molecules will flow around the cannula as in the real animal model), and there will be no absorption inside the syringe.

The results obtained in cat model showed persisting CSF formation (between 3.0 and 5.0 µL/min) even 80 minutes after the animal sacrifice (41). As CSF could not possibly be formed in dead animals, it is possible that a method error is in question. Furthermore, the plastic cylinder model showed that even when the CSF “secretion” was imitated by the infusion of mock CSF into the cylinder with the help of another infusion pump, the calculated V_f_ did not correlate to the secretion result that must be correct as it was “secreted” by the pump, ie, V_f_ = 40.6 ± 1.1 µL/min, but was significantly higher (V_f_ = 46.5 ± 3.2; 41). If this occurred in dead animals and in a plastic cylinder, the question arises as to what exactly the perfusion process does in live animals.

## SOME CONTRADICTIONS IN CALCULATION OF CSF ABSORBTION

As has been mentioned, a false calculation of the CSF formation rate by ventriculo-cisternal perfusion method will result in a false calculation of CSF absorption rate (equation 5). In other words, any criticism of V_f_ is simultaneously a criticism of V_a,_ and all that has been said for CSF formation can be also said for CSF absorption. Nevertheless, it is necessary to comment on illogical and hardly explicable experimental V_a_ results obtained using ventriculo-cisternal perfusion and the equation derived from Heisey et al (21).

**Figure 4:**
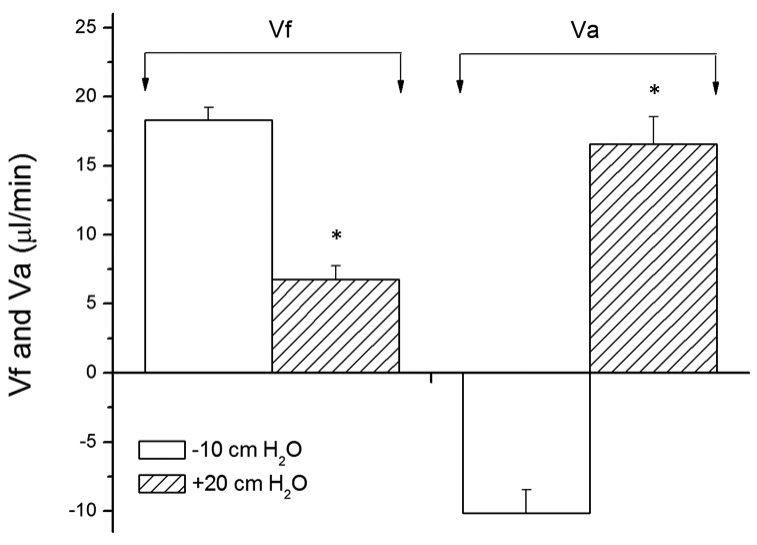
The calculated rate of formed (V_f_) and absorbed (V_a_) CSF during ventriculo-cisternal perfusion with blue dextran at negative (-10 cm H_2_O; empty columns) and positive (+20 cm H_2_O; striped columns) hydrostatic pressure. Each empty column represents the mean value of 16 and a striped column of 15 outflow samples from three cats. The vertical lines represent the standard error of the mean (*P < 0.001; from Orešković) (50)

The results of V_f_ and V_a_ calculation during ventriculo-cisternal perfusion with dextran blue in cats at negative (-10 cm H_2_O) and positive hydrostatic pressure (+20 cm H_2_O) are shown in Figure 4. Elevation of hydrostatic pressure from -10 to +20 cm H_2_O resulted in a substantial V_f_ decrease and V_a_ increase. V_f_ changes related to the hydrostatic pressure are described above, but it is interesting to notice that at negative pressure (-10 cm H_2_O), negative CSF absorption was obtained (-V_a_; -9.8 µL/min). The exact meaning of this result from a pathophysiological point of view is not clear, except that it is a possible artifact of the method. It should be emphasized that the same results (negative value for CSF absorption; -V_a_) were obtained in other studies (18,19,42,43), but they were neither discussed nor explained.

Furthermore, according to the classic hypothesis, there must be a hydrostatic pressure gradient between CSF pressure and blood pressure inside the venous sinuses for passive CSF absorption to occur. Absorption inside the CSF system takes place only when the CSF pressure is higher than 7 cm H_2_O, ie, under this pressure the CSF absorption stops (2,18,19). However, in some experiments V_a_ has been calculated in different animals under hydrostatic pressures at which passive absorption of CSF into the venous blood would be impossible (44-46). These results of CSF absorption, calculated using ventriculo-cisternal perfusion method, cannot be explained by or fitted into the classic hypothesis of CSF physiology, thus discrediting not only the method and the equation for calculating V_a_, but also the classic hypothesis itself.

To summarize, if we are to obtain a correct CSF formation rate (V_f_) by using the equation of Heisey et al (21) it is of utmost importance that CSF is secreted only inside the ventricles and absorbed only outside of them, and that the concentration of marker substance can be changed only by newly formed CSF. However, by using this kind of indirect method it can be demonstrated that CSF is formed and absorbed in all parts of the CSF system, and that the change of marker substance concentration is not only caused by “newly formed CSF.” Namely, marker dilution could additionally be a consequence of the marker substance absorption into the surrounding ventricular tissue, as well as of an inflow (10,47) or outflow (14) of water from the brain ventricles. It could also be a result of different distribution of marker substance throughout the CSF system (Figure 2). All this, together with the calculated negative results for CSF absorption (-V_a_) and a significant CSF absorption rate in conditions where CSF absorption should not exist, seriously discredits ventriculo-cisternal perfusion and the equations for V_f_ and V_a_ calculation as a method that can reliably represent CSF formation and absorption.

## WHAT IS REALLY MEASURED IN CSF SYSTEM BY PERFUSION METHOD?

**Figure 5:**
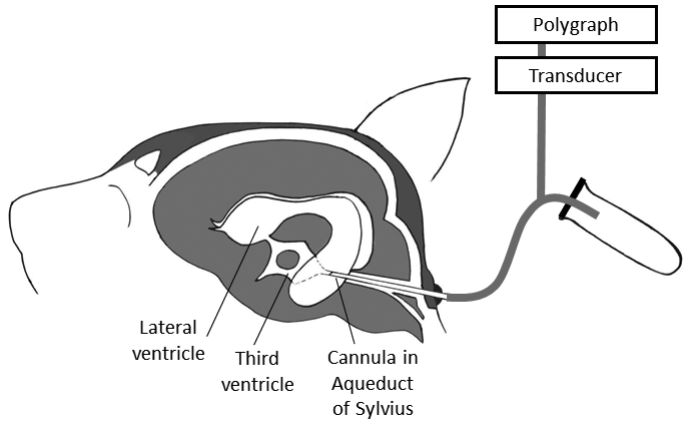
The new experimental method for direct (visual) measurement of cerebrospinal fluid (CSF) formation in cats. The cannula placed into the tunnel inside the cerebellar vermis and into the aqueduct of Sylvius is hermetically closed and used for spontaneous CSF collection at physiological CSF pressure.

In order to explore if CSF formation is actually measured inside the CSF system, we had to take another approach, which better corresponds to the physiological conditions than the classic hypothesis. We introduced a new model, which allowed us to directly observe the CSF volume formation if it really exists. Therefore, in anesthetized cats the cannula is introduced through a tunnel made inside the cerebellar vermis and into the aqueduct of Sylvius, which opens into the IV ventricle. Then the system is hermetically closed to prevent CSF leakage and the influence of atmosphere (Figure 5) (9,15). The external orifice of the cannula is set at the physiologic pressure level, ie, at the value of CSF pressure measured in the CM through the skin, in a cat sphinx position just before any surgical treatment (around 8 cm H_2_O). If we take into account that CSF is secreted predominantly inside the brain ventricles and absorbed predominantly in the subarachnoid space, it is reasonable to assume that under normal CSF pressure it should circulate through the aqueduct of Sylvius, and in our model, through the plastic cannula positioned in the aqueduct. This means that CSF outflow through the external cannula adjusted to the normal CSF pressure values should visually have the direction that corresponds to the CSF flow direction from the ventricles to the subarachnoid space. CSF formation rate is calculated by dividing the collected CSF volume with the time of collection. In this way artificial circulation (in the case of ventriculo-cisternal perfusion caused by the rate of infusion pumps; Figure 1) is avoided, and there is no interference with the possible native CSF circulation rate inside the CSF system. In our experiments, close attention has been paid that CSF collection is carried out under physiological CSF pressure. Therefore, collection process is started only after the cannula has been filled by the fluid and physiological CSF pressure (around 8 cm H_2_O) has been achieved. However, during the collection time of 1, 2, or 3 hours no CSF was observed running out from the system. Only small pulsatile movements of the CSF at the end of the cannula were detected (15,48). In other words, this direct method requires no postulates to be satisfied, no marker substance, no equations for V_f_ calculation, no interference with native CSF circulation and it shows that there is no net CSF formation and unidirectional CSF flow (circulation). This model was tested by using a pump that infused artificial fluid into the LV (imitation of “CSF secretion”), which showed that the volume of infused fluid can be accurately measured. In other words, the exact fluid volume that was infused into the LV was collected into the test tube through the outflow cannula (Figure 5) (15).

## CONCLUSION

Finally, we can conclude that ventriculo-cisternal perfusion is neither a precise nor physiological method for studying cerebrospinal fluid formation and absorption rate. Primarily, the postulates necessary for calculation using the equation of Heisy et al (21) are not satisfied. The method is indirect because it is based on the assumption that marker dilution in perfusate occurs due to CSF secretion within the brain ventricles, and therefore, it is a crucial criterion of CSF formation. Since marker dilution in the CSF system can happen due to many reasons other than the newly formed CSF, the calculated results will be incorrect. The method itself will always give some kind of result by which CSF formation rate can be calculated (when V_f_ is studied in dead and live animals in *in vitro* experiments, during perfusion of any part of the CSF system, etc). If we also take into consideration the results obtained by the direct method, which showed that there was no net CSF formation and circulation, it can be concluded that ventriculo-cisternal perfusion method does not represent the CSF formation and absorption rate. In other words, ventriculo-cisternal perfusion is not a method that will give reliable answers about the existence of CSF formation and absorption.

All of this is in accordance with our new hypothesis (9-12) on CSF homeostasis/physiology, which proposes that CSF is produced and absorbed throughout the entire CSF system, and that the brain and spinal cord perivascular spaces and capillary network play a critical role in the filtration and reabsorption of water volume (9-12,17,49). In other words, there is a permanent fluid and substance exchange between the CSF system and the surrounding tissue, depending on the pathophysiological conditions that predominate within these compartments.
